# Traumatic Rupture and Herniation of the Peroneus Tertius Muscle Leading to Compartment Syndrome and Entrapment of the Superficial Peroneal Nerve: A Case Report

**DOI:** 10.1055/s-0041-1731423

**Published:** 2021-07-19

**Authors:** Eetu N. Suominen, Henrik Sandelin, Jani Puhakka, Jussi Repo, Mikko Ovaska

**Affiliations:** 1 Department of Clinical Medicine, University of Turku, Turku, Finland; 2 Department of Orthopaedics and Traumatology, Helsinki University Hospital and University of Helsinki, Helsinki, Finland; 3 Department of Orthopaedics, Liverpool Hospital, Sydney, New South Wales, Australia; 4 Department of Surgery, Tampere University Hospital, Tampere, Finland; 5 Sports Clinic and Hospital, Pihlajalinna Dextra, Helsinki, Finland

**Keywords:** peroneus tertius, superficial peroneal nerve, compartment syndrome, nerve, entrapment, herniation, anatomical variation, fasciotomy

## Abstract

We present a patient with compartment syndrome and entrapment of the superficial peroneal nerve due to a direct hit to the lateral part of the right lower extremity. The diagnosis of evolving compartment syndrome was made without delay and the patient was quickly taken to the operating theater. Intraoperatively, the entrapment of the superficial peroneal nerve caused by rupture and herniation of the peroneus tertius muscle was surprisingly observed at the site, where the nerve pierces the anterior compartment. The nerve was successfully released in conjunction with fasciotomies of the anterior and lateral compartments. Meticulous diagnosis of compartment syndrome is critical to prevent ischemic injury to muscles and nerves. Recognition of anatomy and anatomical variations is important to prevent iatrogenic injury in unusual circumstances.

The peroneus tertius (PT) muscle is located in the anterior compartment of the leg and produces dorsiflexion and eversion of the foot. The PT muscle is highly prevalent in humans with the prevalence of 80 to 93%. 1 The superficial peroneal nerve (SPN) typically pierces the crural fascia from the lateral compartment, but approximately in one-third of the cases, the fascial opening of the nerve is located in the anterior compartment, as in our case. 2 Traumatic rupture of the PT muscle causing compartment syndrome of leg and entrapment of the SPN is rare; to the best of our knowledge, a similar case has not been previously reported. This case report describes a rupture and herniation of the PT muscle caused by relatively low energy trauma, which in turn led to pressure rise in the anterior and lateral compartments of leg and entrapment of the SPN. The patient provided consent concerning that this case would be submitted for publication.

## Case Report

A 26-year-old healthy male patient presented to a private sports clinic with severe lower-extremity pain. The patient had accidentally been kicked to the lateral aspect of the right lower leg during an indoor soccer game. After the accident, he developed moderate pain to the distal tibia above the talocrural joint line. Radiographs showed no sign of fracture, an observational treatment was selected, and the patient was discharged. During the same evening, the patient returned to the clinic with increased pain and paresthesia at the dorsum of the foot. On clinical examination, the patient's pain had progressed from moderate to severe pain and he had therefore difficulties to move his ankle. Passive range of motion of the ankle was normal but caused pain. Passive flexion of the right hallux generated pain as well. The painful area presented a palpable and visible soft tissue mass. Tinnel's sign was positive, a light tapping over the mass elicited a tingling and painful sensation to the dorsum of the foot. Proximal to the mass at the affected tibiae, there was no pain or numbness. The skin sensation was diminished from the dorsum of the foot to the tip of the dorsum of the hallux. Arteria tibialis posterior and arteria dorsalis pedis were palpable and the capillary reaction in the toe pulps was within 1 second. A pseudoaneurysm was excluded with Doppler ultrasound evaluation. The patient had disproportionate pain and a distinct paresthesia at the dorsum of the foot, that is, a clinical picture suggestive to compartment syndrome.

**Fig. 1:**
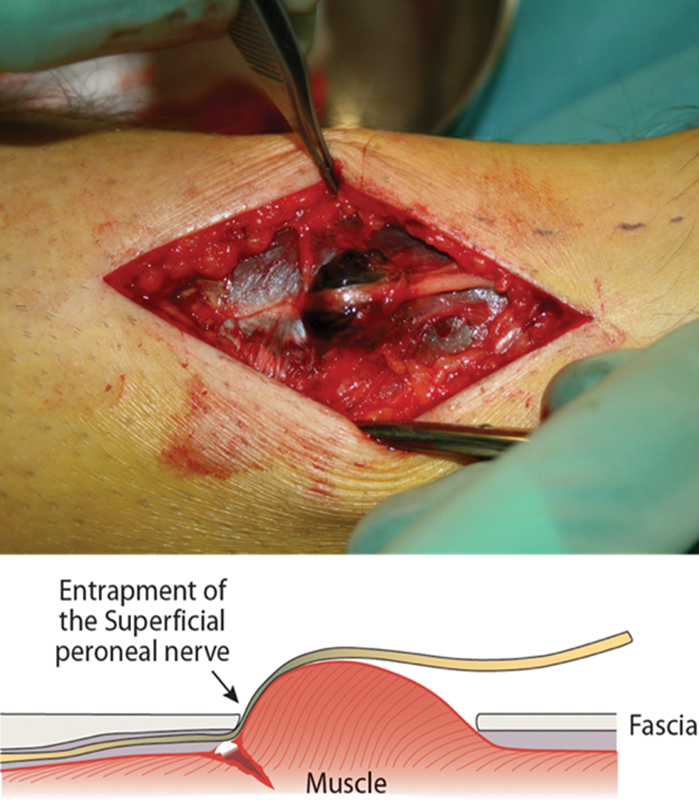
A kick to the lateral aspect of the distal leg during an indoor soccer game caused a rupture and herniation of the peroneus tertius muscle, leading to an entrapment of the superficial peroneal nerve against its fascial opening, and compartment syndrome of the anterior compartment of the leg.

**Fig. 2:**
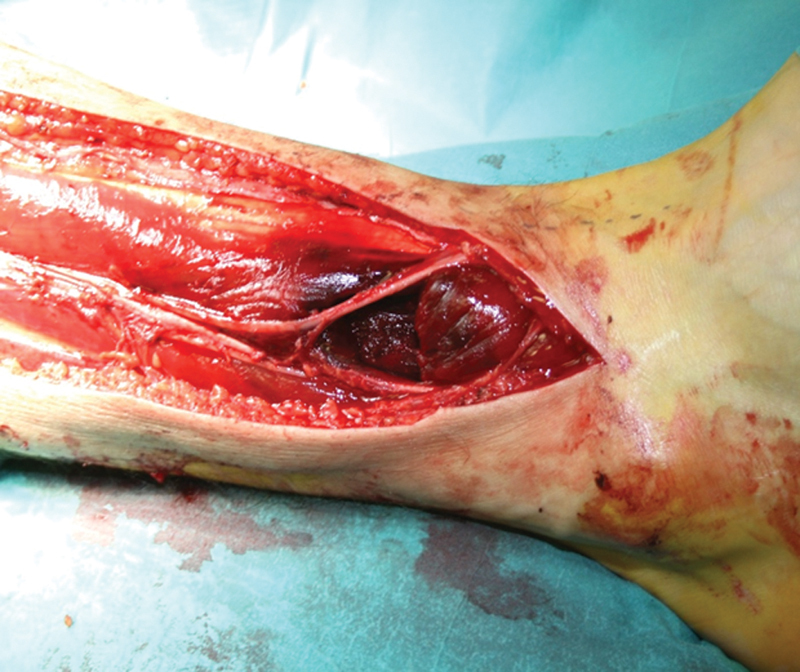
Due to compartment syndrome, full-length fasciotomy of the anterior and peroneal compartments was performed. The peroneal nerve had a slight hourglass-shape at the site of the entrapment. The muscle belly of the peroneus tertius muscle was totally ruptured.

The patient was immediately taken to the operating room for anterior and lateral compartment fasciotomy of the leg. For the surgical procedure, the patient was placed in the supine position, the assumed course of SPN was marked, and a single 10-cm longitudinal incision was performed to the level of the muscle fascia. Surprisingly, entrapment of the SPN was observed at the site, where the nerve pierces the anterior compartment ( Fig. 1 ). The nerve was successfully released in conjunction with fasciotomies of the anterior and lateral compartments. Within the muscle compartment, a hematoma was observed, and the belly of the PT muscle was totally ruptured and herniated ( Fig. 2 ). During the operation, the muscles of the traumatic area recovered exceedingly well, which was observed with improvements in the color and contraction responsiveness. Hemostasis was controlled, wound was rinsed, and packed with sterile dressings. A crepe bandage was applied and finally the ankle was placed in an angle of 90 degrees angle by using a posterior splint.

In the subsequent operation, the muscles were examined carefully. Tibialis anterior, extensor hallucis longus, and extensor digitorum longus muscles were found to be intact and the ruptured muscle was confirmed to be the PT muscle. Macroscopically, the distal part was vital, and therefore, no resection was performed. The incision was closed with sutures and the ankle was placed in a Walker boot for 6 weeks with full weight bearing. Follow-up examinations were performed after 2, 6, and 12 weeks. At the last follow-up at 3 months postoperatively, the patient had full ranges in plantar flexion and eversion/inversion movements of the ankle. The dorsiflexion of the ankle was 10 degrees decreased compared with the healthy side. No deformity was present, although some wasting persisted in the area, observed as a small pit in the ankle. The patient had resumed to physical activity without pain or discomfort. After a follow-up time of 3 years, no new treatments have been needed.

## Discussion

This report describes a rare case of PT muscle rupture causing a compartment syndrome and nerve palsy. Acute limb compartment syndrome (LCS) is a surgical emergency, which is caused by pressure rise within a closed fascial space. In our case, the rupture and herniation of the PT muscle led to pressure rise in the anterior and lateral compartments, which in turn, caused the entrapment of SPN. The entrapment of SPN is a well-known pathology of the lower extremity, with several possible causes. 3 4 The review of the literature suggests that compartment syndrome and entrapment of the superior peroneal nerve caused by traumatic rupture and myofascial herniation of the PT muscle is a rare condition that has not been previously reported. There is, therefore, a lack of information on the clinical significance and therapeutic options for patient with this type of trauma. The most important determinant of a poor outcome from LCS after injury is delay in diagnosis. 5 Pain out of proportion to that expected by the injury should give a cause to suspect LCS. In the present case, a relatively low energy trauma caused increasing pain, paresthesia, and motor weakness with inadequate skin sensation in the dorsum of the foot, which led to suspect LCS and eventually perform emergent surgical treatment.

Both the PT muscle and SPN have clinically and surgically significant variations in morphology and anatomical relationships. The present study emphasizes the knowledge of these variations to interpret symptoms correctly and to avoid iatrogenic injuries during surgery. The PT muscle originates at the anterior distal third of the fibula and courses beneath the superior extensor retinaculum before entering the tendinous sheath of the tibialis anterior. The PT tendon runs obliquely and laterally to the most lateral tendon of extensor digitalis longus muscle and passes beneath the inferior extensor retinaculum before inserting on the dorsal base of the fifth metatarsal. Several reports also support the morphological variation in size, site, insertion, the level of origin, and the number of origins. 6 7 8 9 10 11 Overall, studies and literature of symptomatic PT is scarce and mainly associated with tendon problems, causing catching or locking over the anterolateral ankle or rear-foot and lesions such as split tears or ganglion cysts. 12 13 14 Hypertrophied PT muscle has also been reported to cause snapping and ankle pain. 15

The highly variable anatomical course of the SPN may expose it to damage during surgical incisions over the lateral malleolus, ankle arthroscopy, and forefoot surgery. 16 SPN is the lateral branch of the common peroneal nerve bifurcation and most commonly travels in the lateral compartment of the leg. The nerve courses anteroinferiorly between the peroneus longus, peroneus brevis, and extensor digitorum longus in close proximity to the anterior intermuscular septum. However, alternative routes in (1) anterior compartment, (2) both lateral and anterior compartments, and (3) within the intermuscular septum are known. 17 SPN tends to pierce the crural fascia and become subcutaneous approximately at the distal third of the leg, after which it continues to run superficially over the extensor retinaculum and bifurcates into two exclusively sensorial terminal branches, intermediate and medial dorsal cutaneous nerves, supplying the foot. The SPN is susceptible to mechanical compression along the subfascial course or at the opening where it penetrates the crural fascia. The incidence of nerve compression of SPN has been assumed to be higher than presented in the literature. 19 Compression mechanisms of SPN include fascial entrapment, bony fractures, forced inversion, and lipoma. 20 21

## Conclusion

In conclusion, this is the first report to define the herniation of the PT muscle after a low-energy trauma as a cause of compartment syndrome and entrapment of the SPN. The authors present an effective and safe technique for surgical management as well as postoperative care. It is important to suspect compartment syndrome even after low energy trauma to the leg if the patient presents with pain that is disproportionate to the initial injury. For orthopedic surgeons, this case underlines the importance of knowledge of the anatomical relationship between nerves and muscles. Recognition of common anatomical variations is important to be able to interpret symptoms correctly and to avoid iatrogenic injuries during surgical procedures.
